# Novel Early Pregnancy Multimarker Screening Test for Preeclampsia Risk Prediction

**DOI:** 10.3389/fcvm.2022.932480

**Published:** 2022-07-27

**Authors:** Kaspar Ratnik, Kristiina Rull, Oliver Aasmets, Triin Kikas, Ele Hanson, Kalle Kisand, Krista Fischer, Maris Laan

**Affiliations:** ^1^Department of Biomedicine, Institute of Biomedicine and Translational Medicine, University of Tartu, Tartu, Estonia; ^2^SYNLAB Eesti OÜ, Tallinn, Estonia; ^3^Women's Clinic, Tartu University Hospital, Tartu, Estonia; ^4^Department of Obstetrics and Gynaecology, Institute of Clinical Medicine, University of Tartu, Tartu, Estonia; ^5^Estonian Genome Centre, Institute of Genomics, University of Tartu, Tartu, Estonia; ^6^Department of Internal Medicine, Institute of Clinical Medicine, University of Tartu, Tartu, Estonia; ^7^Institute of Mathematics and Statistics, University of Tartu, Tartu, Estonia

**Keywords:** Preeclampsia, early pregnancy, biomarkers, PTX3, multiplex immunoassay, risk prediction, statistical modeling, 6PLEX

## Abstract

Preeclampsia (PE) is a common pregnancy-linked disease, causing preterm births, complicated deliveries, and health consequences for mothers and offspring. We have previously developed 6PLEX, a multiplex assay that measures PE-related maternal serum biomarkers ADAM12, sENG, leptin, PlGF, sFlt-1, and PTX3 in a single test tube. This study investigated the potential of 6PLEX to develop novel PE prediction models for early pregnancy. We analyzed 132 serum samples drawn at 70–275 gestational days (g days) from 53 pregnant women (PE, *n* = 22; controls, *n* = 31). PE prediction models were developed using a machine learning strategy based on the stepwise selection of the most significant models and incorporating parameters with optimal resampling. Alternative models included also placental *FLT1* rs4769613 T/C genotypes, a high-confidence risk factor for PE. The best performing PE prediction model using samples collected at 70–98 g days comprised of PTX3, sFlt-1, and ADAM12, the subject's parity and gestational age at sampling (AUC 0.94 [95%CI 0.84–0.99]). All cases, that developed PE several months later (onset 257.4 ± 15.2 g days), were correctly identified. The model's specificity was 80% [95%CI 65–100] and the overall accuracy was 88% [95%CI 73–95]. Incorporating additionally the placental *FLT1* rs4769613 T/C genotype data increased the prediction accuracy to 93.5% [AUC = 0.97 (95%CI 0.89–1.00)]. However, 6PLEX measurements of samples collected at 100–182 g days were insufficiently informative to develop reliable PE prediction models for mid-pregnancy (accuracy <75%). In summary, the developed model opens new horizons for first-trimester PE screening, combining the easily standardizable 6PLEX assay with routinely collected antenatal care data and resulting in high sensitivity and specificity.

## Introduction

Preeclampsia (PE) is considered a disease of the placenta, caused by impaired remodeling of spiral arteries during the first half of pregnancy and/or suboptimal placental capacity to support the maternal-fetal needs until natural delivery. As a result of insufficient modulation of the uterine vasculature in the beginning of the pregnancy, hypertension (HTN) and other signs of organ dysfunction, the characteristics of PE, manifest during the second half of pregnancy (1, 2). This is mediated by hypoxic placenta releasing biomolecules into maternal circulation that causes endothelial damage and generalized inflammatory vascular stress. PE is a sudden and severe complication with a prevalence of 4.6% worldwide and in Europe alone affecting annually ~400,000 cases. Currently, the most effective management of PE is delivery that can occur preterm. PE can also cause fetal growth restriction or even intrauterine or maternal death in extreme cases (3). In addition, hypertensive pregnancy complications result in a greater risk of developing cardiovascular and metabolic diseases, stroke, and end-stage renal disease later in life (4).

It has been recently shown that low-dose aspirin has a protective effect in the prevention of PE in high-risk cases (up to 82% for early PE, onset before 34 gestational weeks (g week)) if prophylaxis is started before g week 16 (5, 6). The development of effective screening tools for PE to be applied in the 1st trimester of pregnancy has been sought for and advised by professional organizations (4, 7–9). Traditionally, evaluation of PE risk is based on the assessment of maternal prepregnancy characteristics, such as maternal age >40 years, nulliparity, obesity, previous or family history of PE, diabetes mellitus, chronic HTN, and renal or autoimmune diseases. Although it is a maternal risk factor-based model that is easy to perform, it has a low detection rate (~40%) for the subsequent manifestation of PE (10). Therefore, the application of other parameters to be measured during pregnancy has been proposed for the estimation of the PE risk. These include mean arterial pressure (MAP) and uterine arteries pulsatility index (UtA PI), a single or combination of biochemical serum markers (11). The predictive rate of these algorithms has been reported up to 96.3% [false-positive rate (FPR) 10%] for early PE with the onset before 34th g week, but remains lower (up to 76.6%) for late PE that represents the major fraction of PE cases (12, 13). The modest prediction rate of late PE has limited the investigation of its early preventive measures. For example, pravastatin, administered after 35–37 g week for high-risk women, did not prevent the manifestation of disease (14). It indicates either the ineffectiveness of pravastatin or suboptimal selection of high-risk patients destined to develop term PE.

We have recently developed an innovative, single-tube multimarker 6PLEX assay for the Luminex^®^ xMAP platform to measure simultaneously six PE-related biomarkers in the sera of pregnant women, namely, soluble fms-like tyrosine kinase 1 (sFlt-1), placental growth factor (PlGF), soluble endoglin (sENG), leptin, disintegrin, and metalloproteinase domain-containing protein 12 (ADAM12), and Pentraxin 3 (PTX3) (15). Combining 6PLEX measurements of serum samples collected in the second half of pregnancy with gestational age and maternal weight at sampling generated a clinically applicable and effective prediction formula for PE development regardless of gestational age at its clinical manifestation. The new solution exhibited superior prognostic yield compared to the currently used sFlt-1/PlGF ratio in the diagnosis confirmation of symptomatic women in late pregnancy (96.5% vs. 73.7%) (15, 16).

This study aimed to investigate the potential of 6PLEX to develop novel PE prediction models for early pregnancy. We analyzed serum samples from pregnant women drawn during either 70–98 or 100–182 gestational days (g days), including cases who later experienced PE and controls with an uneventful pregnancy until delivery. Combining biomarker measurements with maternal data enabled the generation of new PE prediction models with high specificity and sensitivity to correctly identify PE onset many months later. Innovatively, the developed 1st trimester PE prediction models were further improved by incorporating the placental genotypes of the genetic variant rs4769613 T/C. This variant is localized upstream of the *FLT1* gene, and the carriership of the C-allele, especially CC-homozygosity, represents a high-confident genetic risk factor for late-onset PE (17, 18).

## Methods

### Clinical Study Material

The study patients were recruited, and the clinical data and biomaterials were collected during a prospective observational “Happy Pregnancy” project (full name: “Development of novel non-invasive biomarkers for fertility and healthy pregnancy', PI: M.L.). The project has been approved by the Ethics Committee of Human Research of the University Clinic of Tartu, Estonia (permission no. 221/T-6, 17.12.2012, and 286/M-18, 15.10.2018) and was carried out in compliance with the Helsinki Declaration. Written informed consent to participate in the study was obtained from everyone prior to recruitment. All participants were of white European ancestry and lived in Estonia.

The pregnant women had been enrolled at their first antenatal visit at the Women's Clinic, Tartu University Hospital, Estonia in 2013–2015. The pregnancy follow-up was based on the national guidelines of the Estonian Gynaecologists' Society (19). The collected clinical and epidemiological data are specified in the Supplementary Methods. The diagnosis of PE followed the international guidelines at the time of recruitment and included co-occurrence of a new-onset HTN (blood pressure ≥140/ ≥90 mmHg) after 20 g week and proteinuria (PTN), or other signs of maternal organ dysfunction (3). Diagnosis of small-for-gestational-age (SGA) newborn was assigned at the delivery based on national guidelines (20).

For research purposes, serum samples were collected in parallel with blood samples for routine clinical tests. Each Happy Pregnancy study subject had been drawn a blood sample 2–5 times during the pregnancy. Serum was separated (centrifugation at 1,800 g for 10 min at room temperature, RT) within 2 h after sampling and kept at −80°C before further aliquoting and subsequent analysis.

Preeclampsia patients (*n* = 22, age 28.0 ± 5.2 years; prepregnancy body mass index (BMI) 26.8 ± 6.2 kg/m^2^) and non-PE controls (*n* = 31; 28.5 ± 5.1 years; BMI 25.7 ± 4.8 kg/m^2^) analyzed in this study were selected from the Happy Pregnancy biobank based on available gestational-age matched serum samples in both, PE case and control groups (Supplementary Table S1). The included pregnancies had been drawn from a total of 132 serum samples (Table 1). Early pregnancy was represented by 14 samples from women who later developed PE (drawn 88.8 ± 7.0; 70–98 g days) and 20 samples from healthy gestations (88.2 ± 5.8; 76–96 g days). Within this time window, one PE pregnancy and two control cases were represented by two sera, drawn at 70th and 96th, 76th and 92nd, 90th, and 93rd g days, respectively. Mid-pregnancy sample set comprised 18 PE and 21 non-PE pregnancy sera, sampled between 100–180 (150.7 ± 25.7) and 109–182 (136.1 ± 24.1) g days, respectively. During mid-gestation, one woman in the PE and three in the control group had been sampled two times, at 117th and 180th, 111th and 182nd; 118th and 139th; and 121st and 147th g days, respectively. All 53 women, irrespective of the final pregnancy outcome (PE or non-PE), were normotensive at blood draw during early and mid-pregnancy blood sampling. The age at the onset of PE in cases with early- and mid-pregnancy serum samples was 257.4 ± 15.2 and 249.3 ± 25.3 g days, respectively.

**Table 1:** Maternal and pregnancy characteristics of the cases involved in PE prediction modeling.

	**Early pregnancy samples**		**Mid-pregnancy samples**	
	**Control**	**Later PE**	**Control**	**Later PE**
**Sampling data**				
Number of serum samples	20	14	21	18
Gestational age at sampling (g days)	88.2 ± 5.8	88.8 ± 7.0	136.1 ± 24.1	150.7 ± 25.7
Maternal weight at sampling (kg)	74.3 ± 15.5	70.2 ± 15.1	79.5 ± 15.4	77.1 ± 15.5
**General data of the index pregnancy**				
Number of cases	18	13	18	17
Maternal age (years)	26.8 ± 4.6	26.6 ± 3.7	29.3 ± 6.4	28.1 ± 5.5
Nulliparity (n, %)	8 (44.4%)^*^	12 (92.3%)	10 (55.5%)	13 (76.5%)
Gravidity (n)	1.8 ± 1.0	1.4 ± 0.9	2.2 ± 1.6	1.6 ± 1.2
Pre-pregnancy BMI (kg/m2)	25.3 ± 4.7	24.2 ± 4.4	26.1 ± 5.0	27.0 ± 7.0
Fetal sex (male/female)	10/ 8	7/ 6	9/ 9	11 / 6
Diagnosis of preeclampsia (g days)	n.a.	257.4 ± 15.2	n.a.	249.3 ± 25.3
Gestational diabetes (n, %)	2 (11.1%)	0 (0%)	2 (11.1%)	0 (0%)
Gestational age at delivery (g days)	278.6 ± 13.0	260.4 ± 16.2	278.4 ± 13.3	257.5 ± 22.2
Preterm delivery, <259 g days (*n*, %)	1 (5.5%) ^*^	5 (38.4%)	1 (5.5%) ^*^	9 (52.9%)
SGA^a^ newborn (*n*, %)	1 (5.5%) ^*^	6 (46.2%)	2 (11.31%) ^*^	8 (47.1%)
Placental genotype for rs4769613 (CC/CT/TT) ^b^	4/9/4	4/6/1	6/8/2	4/7/2

*Data are given as either mean ± standard deviation or number (%) for the continuous or categorical variables, respectively*.

a *Diagnosis of a small-for-gestational-age (SGA) newborn was assigned at the delivery based on national guidelines (20)*.

b *Placental tissues were available for genotyping for 17 of 22 PE and 29 of 31 no-PE cases included in the study (in total 46 of 53 pregnancies); placental genotype of a single-nucleotide variant rs4769613 T/C represents a risk factor for late-onset PE as it is localized in an enhancer region near the FLT1 gene, modulating gene expression (17, 18)*.

* *P < 0.05 between PE and no-PE groups; categorical variables, chi-square test, non-categorical variables Wilcoxon rank-sum test*.

*BMI, body mass index; g days, gestational days; gravidity, the total number of pregnancies including index pregnancy; n, number of subjects; nulliparity, no previous deliveries; PE, preeclampsia; n.a., not applicable*.

Late pregnancy serum samples of these 53 cases (28 PE, 206–275 g days; 31 controls, 210–274 g days) have been utilized in our recent study reporting PE prediction models based on 6PLEX assay measurements of late pregnancy serum samples (15). Two serum samples (one PE and one control, 182 and 180 g days, respectively) from this seminal publication were reallocated in this study to the mid-pregnancy sample set, as more appropriate.

### Biomarker Measurements Using the Luminex^®^ 6PLEX Multiplex Assay

The Luminex^®^ xMAP platform offers advanced immunoassay-based technology that allows rapid simultaneous analyses of a large number of biomarkers in a single test tube (21). Luminex^®^ xMAP-based approach and development of the methodology for multiplex measurement of sFlt-1, PlGF, sENG, leptin, ADAM12, and PTX3 in a single test tube is detailed in a previously published study (15). Briefly, Luminex^®^ magnetic microspheres (#MC100) and antibody coupling kit for covalent linking of antibodies and microspheres (Antibody Coupling Kit, #40–50016) were purchased directly from Luminex^®^ Corporation (Austin TX, USA). Capture and detection antibodies and reference proteins were purchased from R&D Systems (Minneapolis, MN, USA) (Supplementary Table S2). The applied Luminex^®^ sandwich immunoassay protocol followed the manufacturer's guidelines (The xMAP Cookbook, https://www.luminexcorp.com/). All serum samples were analyzed in one batch in duplicate using a 1:20 dilution factor. All dilutions of reference proteins and tested samples were prepared in General Assay Diluent (GAD; #620; ImmunoChemistry Technologies, LLC, Minnesota, USA). Details of Luminex^®^ xMAP technology used equipment, reagents, and their dilutions are provided in the Supplementary Methods and our seminal methodological publication (15).

### Placental *FLT1* rs4769613 Genotyping

Placental tissues were collected after cesarean section or vaginal delivery, were washed with 1 × PBS (phosphate-buffered saline, pH~7.4) to remove maternal blood, and stored in a dry tube at −80°C until DNA extraction. DNA from the placental samples was extracted using the NucleoSpin Tissue kit (MACHEREY-NAGEL GmbH & Co. KG, Düren, Germany) according to the manufacturer's protocol. For genotyping of placental rs4769613 T/C, premade TaqMan Genotyping Assay was used according to the manufacturer's protocol (Applied Biosystems, Foster City; Assay ID: C__32231378_10). Placental tissue was available for 17 of 22 PE cases and 29 of 31 controls (in 46 of 53 pregnancies). Genotyping details have been described in the Supplementary Methods and a recent publication (18).

### Statistical Analysis and PE Prediction Algorithm Development

Summary estimates of the data were calculated, and all statistical tests were implemented using the STATA software version 13.1 (StataCorp, TX, USA) or the R 3.3.3 language and environment (Free Software Foundation, Boston, MA, USA, http://www.r-project.org). To compare groups, the Mann-Whitney rank-sum test was used for continuous variables and the chi-square test for categorical variables. *P* < 0.05 was considered statistically significant.

Logistic regression models (GLM) were used to investigate associations between biomarker measurements and the clinical onset of PE during the index pregnancy. All biomarker values were centered and scaled before modeling for data normalization and standardization. First automated computational pre-filtration for the identification of the optimal prediction model has performed modeling with the stepAIC selection method (generalized linear model with stepwise feature selection) in package CARET (short for **C**lassification **A**nd **RE**gression **T**raining). This machine learning strategy, in combination with leave-one-out cross-validation (LOOCV), allows to select statistically most significant prediction model and to pick the complexity parameters that are associated with the optimal resampling statistics. Pre-filtration was carried out by using the following input variables: measured concentrations of six biomarkers (ADAM12, Leptin, Pentraxin3, sENG, sFlt-1, and PlGF) and maternal characteristics of blood sampling time in gestational days, maternal weight at blood draw, and parity as a binary variable (nulliparity defined as 0 and multiparity defined as 1). As an output of this procedure, all possible models generated from these input parameters were automatically ranked based on their area under the curve (AUC) estimates. The best-predicted model by the LOOCV + stepAIC approach was developed further by alternatively replacing and/or adding biomarker measurements to trial the model performance using the GLM package in R. Additionally, statistical models were built combining parameter combinations with the placental genotypes of the single-nucleotide polymorphism (SNP) rs4769613 T/C (in an additive manner, defined as variables 0, 1, or 2 according to genotypes TT, CT, and CC) either by replacing parity with the SNP data or considering them both.

The predictive power of the models was assessed using the ROC curve (receiver operating characteristic curve) and the area under the ROC curve (AUC). For every fitted model, model-based individual predictions were obtained, as estimated probabilities of PE (during the index pregnancy until term), denoted as p(i) with Epi::ROC. The p(i) represents the probability thresholds at the maximum Youden's J index on the curve. The p(i) equal or superior to a fitted model optimal cutoff point value indicates that the subject will develop PE or has PE, whereas the p(i) inferior to a fitted model optimal cutoff point value predicts that PE will not develop.

## Results

### Gestational Dynamics of Maternal Serum ADAM12, PTX3, PlGF, sFlt-1, sENG, and Leptin Levels in Healthy and Preeclamptic Pregnancies

**Figure 1:**
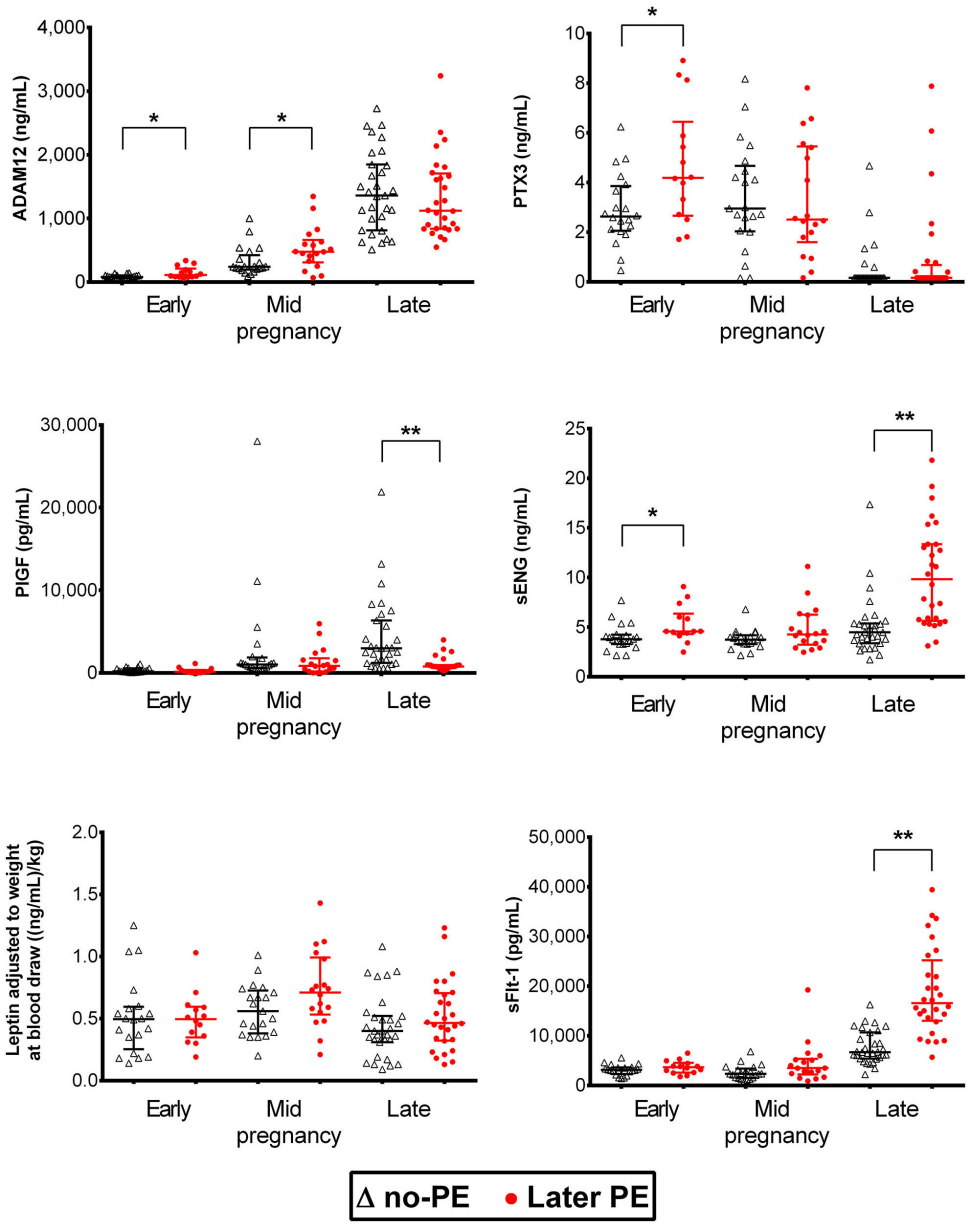
Dynamics of preeclampsia-related biomarkers throughout gestation in healthy and preeclamptic pregnancies. Luminex^®^ 6PLEX assay measurement data of concentrations of ADAM12, PTX3, PlGF, sENG, and sFlt-1 in 132 serum samples collected from pregnant women. The early pregnancy group was comprised of 14 samples drawn at 70–98 g days from women that later developed PE and 20 samples from controls collected at 76–96 g days. The mid-pregnancy sample set comprised 18 PE and 21 control pregnancy sera, sampled at 100–180 and 109–182 g days, respectively. Data of late pregnancy samples representing 28 PE (206–275 g days) and 31 control sera (210–274 g days) were obtained from our recent study (15). All women, irrespective of the pregnancy outcome (PE or no-PE), were normotensive at blood draw. The whiskers on the plot show the median with an interquartile range. The statistical difference in biomarker distributions between PE and control cases was compared using the Mann-Whitney U-test, ^*^ representing *P* < 0.05 and ^**^ *P* < 0.0001. ADAM12, ADAM Metallopeptidase Domain 12; g days, gestational days; PlGF, placental growth factor; PTX3, Pentraxin3; sENG, endoglin; sFlt-1, soluble fms-like tyrosine kinase 1.

**Figure 2:**
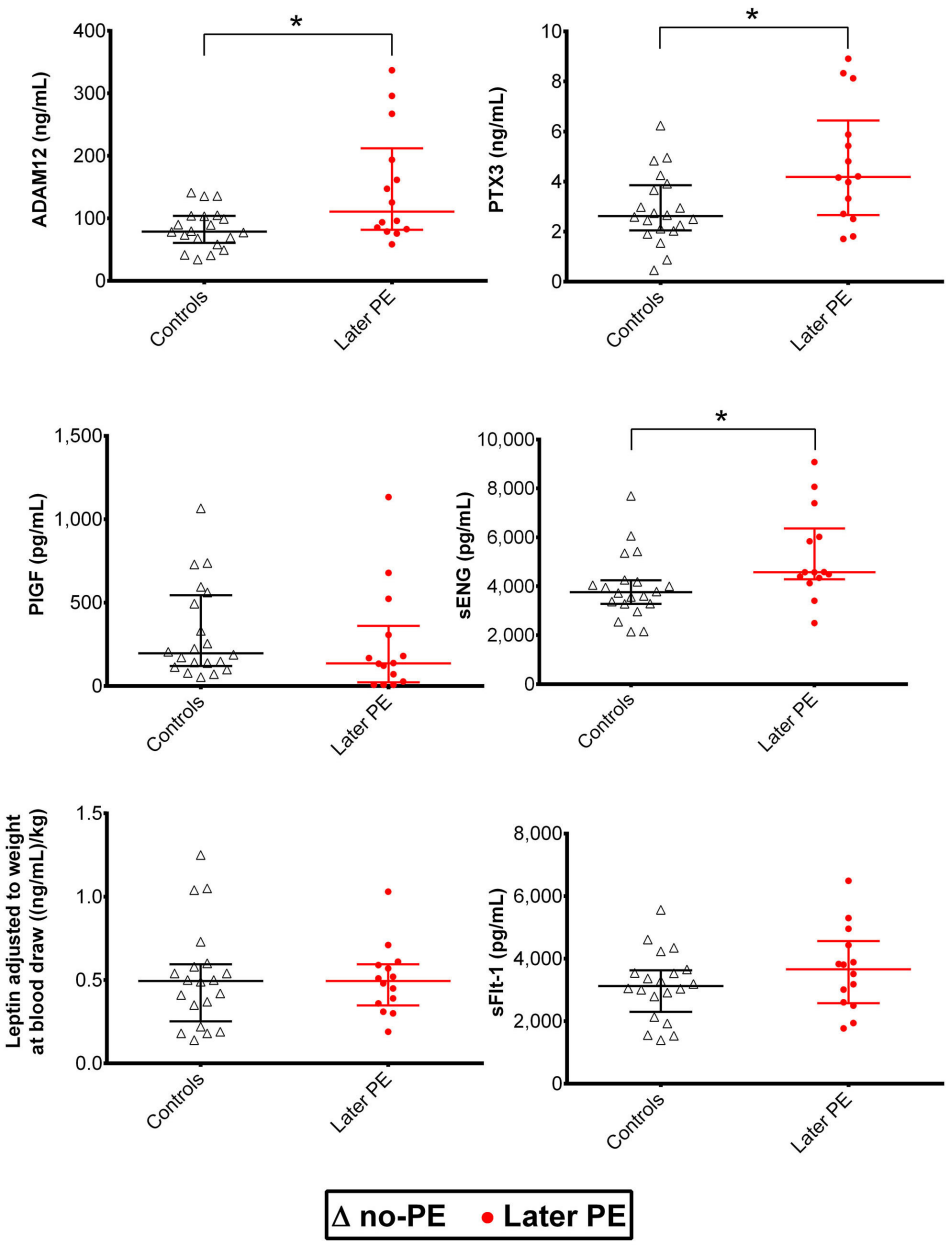
Distribution of maternal serum biomarkers measured with the Luminex^®^ 6PLEX assay during early pregnancy, stratified by the later onset of preeclampsia (PE). Samples from women that later developed PE (*n* = 14, drawn at 70–98 gestational days) were compared to samples representing uncomplicated gestations until term (*n* = 20, 76–96 gestational days; Table 1). The whiskers on the plot show the median with an interquartile range. The statistical difference in biomarker distributions between PE and control cases was compared using the Mann-Whitney U-test, * representing *P*<0.05. ADAM12, disintegrin and metalloproteinase domain-containing protein 12; PlGF, placental growth factor; PTX3, pentraxin-3; sENG, soluble endoglin; sFlt-1, soluble fms-like tyrosine kinase-1.

Luminex^®^ 6PLEX multiplex assay measurements of 132 serum samples (drawn from 53 pregnant women between 70 and 275 g days; Table 1) revealed different gestational dynamics of the analyzed PE-linked biomarkers ADAM12, PTX3, PlGF, sFlt-1, sENG, and leptin (Figure 1, Table 2). In normal pregnancy, ADAM12 and PlGF levels gradually increase through all three trimesters. PTX3 serum concentrations maintained stable levels during early and mid-pregnancy with a significant decrease toward the term. In contrast, both sFlt-1 and sENG have an increasing trend specifically in late pregnancy. During early pregnancy, three biomarkers showed significantly (*P* < 0.05) increased serum levels in cases with a later onset of PE compared to controls: PTX3, ADAM12, and sENG, whereas only ADAM12 maintained higher concentration in the PE group also in mid-pregnancy (Figures 1, 2). In late pregnancy serum samples from future PE cases, increased sENG and sFlt-1, and decreased PlGF were measured (PE vs. non-PE serum levels, *P* < 0.001).

**Table 2:** Measured concentrations of biomarkers in maternal sera sampled in early, mid, and late pregnancy.

	**Early pregnancy**		**Mid pregnancy**		**Late pregnancy^a^**	
	**Controlsn *n* = 20**	**Later PE*n* = 14**	**Controls *n* = 21**	**Later PE *n* = 18**	**Controls *n* = 31**	**Later PE*n* = 28**
Gestational days at sampling (*n*)	88.2 ± 5.8 89 (76–96)	88.8 ± 7.0 89 (70–98)	136.1 ± 24.1 137 (109–182)	150.7 ± 25.7 137 (100–180)	236.2 ± 17.2 230 (210–274)	236.3 ± 20.8234 (206–275)
ADAM12 (ng/mL)	79.1 (69.1–98.5)	110.8^*^ (97.8–202.3)	239.2 (196.6–421.0)	474.6^*^ (249.3–632.3)	1362 (813.4–1847)	1121(837.7–1705)
PTX3 (ng/mL)	2.63 (2.24–3.56)	4.19^*^ (3.33–6.08)	2.95 (2.04–4.67)	2.55 (1.79–5.41)	0.16 (0.16–0.85)	0.16(0.16–1.75)
PlGF (pg/mL)	196.4 (188.1–451.6)	136.10 (63.9–436.5)	1011 (611–1872)	884.1 (184.3–1559)	2974 (1197–6356)	780.1^**^(577–939)
sENG (ng/mL)	3.76 (3.35–4.59)	4.57^*^ (4.18–6.30)	3.74 (3.31–4.17)	4.19 (2.90–6.22)	4.48 (3.37–5.38)	9.82^**^(5.62–13.36)
sFlt-1 (pg/mL)	3119 (2627–3638)	3660 (2895–4422)	2329 (1486–3394)	3472 (2384–5168)	6693 (5408–10686)	16543^**^(13012–25202)
Leptin adjusted to weight ((ng/mL)/kg)	0.49 (0.37–0.66)	0.49 (0.38–0.62)	0.56 (0.38–0.73)	0.69 (0.55–0.98)	0.40 (0.31–0.52)	0.46(0.33–0.71)

*All biomarkers were measured in a single test tube using the Luminex^®^ 6PLEX assay. Biomarker data are presented as median with 95% CI*.

Gestational days are presented as mean ± standard deviation and median (range). The Mann–Whitney U–test for non–categorical variables was applied to compare biomarker levels between groups, ^*^ denotes P < 0.05 and ^**^ P < 0.0001.

a *Data from the study developing PE prognosis models based on 6PLEX assay measurements of late pregnancy serum samples (15)*.

*ADAM12, disintegrin and metalloproteinase domain–containing protein 12; PlGF, placental growth factor; PTX3, pentraxin-3; sENG, soluble endoglin; sFlt-1, soluble fms–like tyrosine kinase-1*.

### Placental Rs4769613 T/C Variant Near *FLT1* Is Not Associated With sFlt-1 Serum Levels

Placental tissues from 46 women (17 PE and 29 non-PE) analyzed in this study were available for placental genotyping of *FLT1* rs4769613 T/C. No statistically significant differences were observed in serum sFlt-1 levels in early, mid, or late pregnancy between women with alternative genotypes CC, CT, and TT (Table 3).

**Table 3:** Detailed data of sFlt-1 (pg/mL) measurements stratified into fetal *FLT1* rs4769613 T/C genotype of the 116 serum samples drawn during early, mid, and late pregnancy.

	**Early pregnancy *n* = 31**	**Mid–pregnancy *n* = 32**	**Late–pregnancy *n* = 53**
g days	90 (70–98)	144 (100–182)	236(206–275)
CC	3531 (2131–6489)	2339 (1309–6489)	11862 (4782–39417)
CT	3191 (1531–5161)	2770 (813–5989)	9158 (2207–34221)
TT	3292 (1396–5298)	4427 (3491–6805)	8758 (4301–29884)

*sFlt-1 (pg/mL) data are presented as medians with minimum and maximum values. Gestational age data in days are presented as median (minimum—maximum)*.

*sFlt-1, soluble fms-like tyrosine kinase-1; g days, gestational age in days*.

### Potential of 6PLEX Assay During Early Pregnancy in Predicting Risk for PE Development

**Figure 3:**
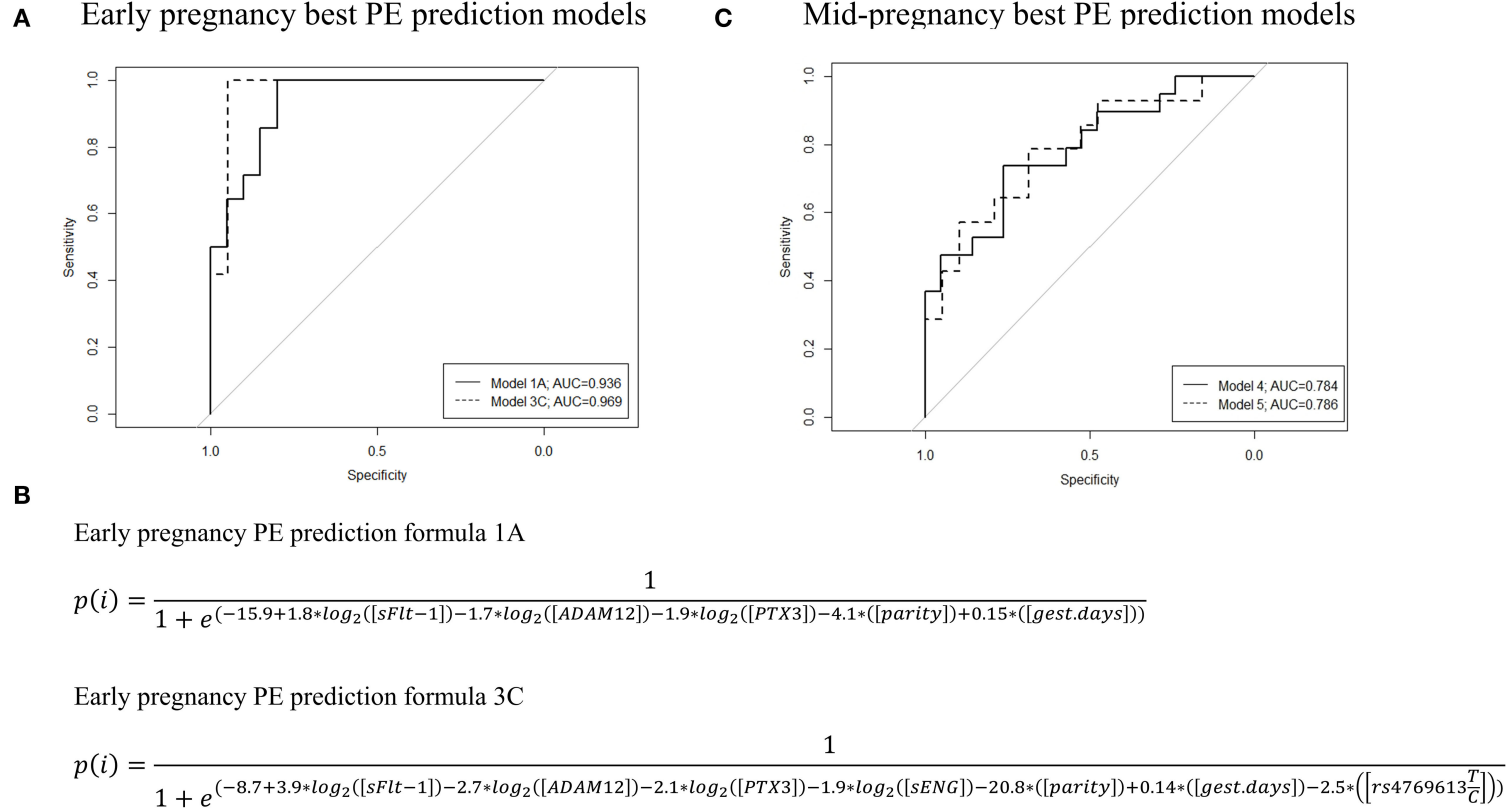
ROC curves, AUC values, and the formulas of the best performing preeclampsia (PE) prediction models were developed based on the Luminex^®^ 6PLEX assay data. The best PE prediction models are based on the analysis of serum samples collected during either **(A)** 70th-98th or **(C)** 100th−182nd g days. Early pregnancy model 1A incorporated data from three biomarkers (sFlt-1, ADAM12, and PTX3) and model 3C four markers (sFlt-1, sEng, ADAM12, and PTX3), whereas mid-pregnancy models 4 and 5 are based on only PlGF measurements. Model 3C and model 5 additionally included placental genotype data of the *FLT1* rs4769613 T/C variant. All models were adjusted for gestational days (gest. days) at sampling and maternal parity (nulliparity or multiparity). **(B)** PE prediction formulae for models 1A and 3C. Details are presented in Tables 4, 5 and Supplementary Table S3. AUC, the area under the curve; ROC, receiver operating characteristics.

Preeclampsia prediction models applicable in early pregnancy were developed by combining Luminex^®^ 6PLEX multiplex-assay measurements of PE biomarkers in maternal serum (70-98 g days) with informative clinical data (Figures 3A,B; Tables 4, 5; Supplementary Table S3). According to the machine-learning approach (LOOCV + stepAIC), the best performing PE prediction model comprised of PTX3, sFlt-1, and ADAM12 measurements, the subject's parity, and gestational age at sampling [model 1A: AUC 0.936 (95%CI 0.843–0.993)]. PTX3 concentration and parity information had a statistically significant contribution to the model (*P* < 0.05). This model correctly “ruled in” or “ruled out” the onset of PE for 30 of 34 analyzed samples (accuracy 88.2%; 95%CI 73.4–95.3). The PE prediction model 1A exhibited 100% sensitivity in identifying all 14 cases who developed PE several months later (onset 257.4 ± 15.2 g days). The specificity of this model was 80.0%. Four of 20 cases progressing healthy pregnancy until delivery received a false-positive outcome regarding the PE prediction.

**Table 4:** Developed PE prognosis models and contributing variables.

**Model acronym**	**Variables contributing to alternative models^a^**						
**Early pregnancy data modeling (70–98th gestational days)**							
1A^b^	PTX3^*^	sFlt-1		ADAM12	parity^*^		g days
1B	PTX3^*^		sENG	ADAM12	parity^*^		g days
1C	PTX3^*^	sFlt-1	sENG	ADAM12	parity^*^		g days
2A	PTX3^*^	sFlt-1		ADAM12^*^		rs4769613 T/C	g days
2B	PTX3^*^		sENG	ADAM12^*^		rs4769613 T/C	g days
2C	PTX3^*^	sFlt-1	sENG	ADAM12^*^		rs4769613 T/C	g days
3A	PTX3	sFlt-1		ADAM12	parity	rs4769613 T/C	g days
3B	PTX3		sENG	ADAM12	parity	rs4769613 T/C	g days
3C	PTX3	sFlt-1	sENG	ADAM12	parity	rs4769613 T/C	g days
**Mid–pregnancy data modeling (100–182th gestational days)**							
4^b^			PlGF		parity		g days
5			PlGF		parity	rs4769613 T/C	g days

a *Parity was treated as a binary variable; every woman was assigned as nulliparous referring to no previous deliveries, or multiparous indicating at least one childbirth before the index pregnancy; rs4769613 T/C refers to the placental genotype of a single–nucleotide variant near the FLT1 gene with T– and C–alleles*.

b *Selected as statistically most significant model using the automatic computational pre–filtration approach (LOOCV + stepAIC)*.

* *p–value <0.05, showing a statistically significant contribution of this variable to the model*.

*ADAM12, disintegrin and metalloproteinase domain–containing protein 12; g days, gestational age in days; PlGF, placental growth factor; PTX3, pentraxin−3; sENG, soluble endoglin; sFlt-1, soluble fms–like tyrosine kinase−1*.

**Table 5:** Characteristics of PE prediction models.

**Model^a^**	**Correct prognosis (accuracy%[95%CI)]**	**AUC [95%CI]**	**Sensitivity % [95%CI]**	**Specificity % [95%CI]**
**Early pregnancy data modeling (70–98th g days) ^b^**				
Models combining biomarkers, gestational age and parity				
1A^c^	30/34 (88.2% [73.4–95.3])	0.936 [0.843–0.993]	100.0 [92.9–100.0]	80.0 [65.0–100]
1B	29/34 (85.29% [69.9–93.6])	0.914 [0.804–0.989]	100.0 [78.6–100.0]	80.0 [60.0–100.0]
1C	30/34 (88.2% [73.4–95.2])	0.932 [0.839–0.993]	100.0 [92.9–100.0]	80.0 [65.0–100.0]
Models combining biomarkers, gestational age and rs4769613 T/C placental genotype				
2A	26/31 (83.9% [67.4–92.9])	0.934 [0.829–1.000]	100.0 [75.0–100.0]	78.9 [68.4–100.0]
2B	25/31 (80.7% [63.7–90.8])	0.930 [0.829–0.996]	91.7 [75.0–100.0]	78.9 [57.9–100.0]
2C	27/31 (87.1% [71.2–94.9])	0.934 [0.825–1.000]	91.7 [83.3–100.0]	89.5 [57.9–100.0]
Models combining biomarkers, gestational age, parity and rs4769613 T/C placental genotype				
3A	29/31 (93.5% [79.3–98.2])	0.969 [0.882–1.000]	100.0 [83.3–100.0]	94.7 [89.5–100.0]
3B	29/31 (93.5% [79.3–98.2])	0.947 [0.851–1.000]	100.0 [83.3–100.0]	89.5 [84.2–100.0]
3C	29/31 (93.5% [79.3–98.2])	0.969 [0.895–1.000]	100.0 [100.0–100.0]	94.7 [89.5–100.0]
**Mid–pregnancy data modeling (100–182**^**th**^ **g days)**				
Model combining PlGF measurement, gestational age and parity				
4^c^	29/39 (74.4% [58.9–85.4])	0.784 [0.634–0.912]	73.7% [36.8–94.7]	76.2% [19.0–85.7]
Model combining PlGF measurement, gestational age, parity and rs4769613 T/C placental genotype				
5	23/32 (71.9% [54.6–84.4])	0.786 [0.613–0.932]	78.6% [28.6–92.9]	68.4% [36.8–100]

a *Detailed information is available in Supplementary Table S3*.

b *Early pregnancy model A includes serum measurements of ADAM12, PTX3, and sFlt–1; model B measurements of ADAM12, PTX3, and sENG, and model C measurements of ADAM12, PTX3, sFlt–1, and sENG*.

c *Selected as statistically most significant model using the automatic computational pre–filtration strategy*.

*AUC, the area under the curve; g days, gestational days*.

**Figure 4:**
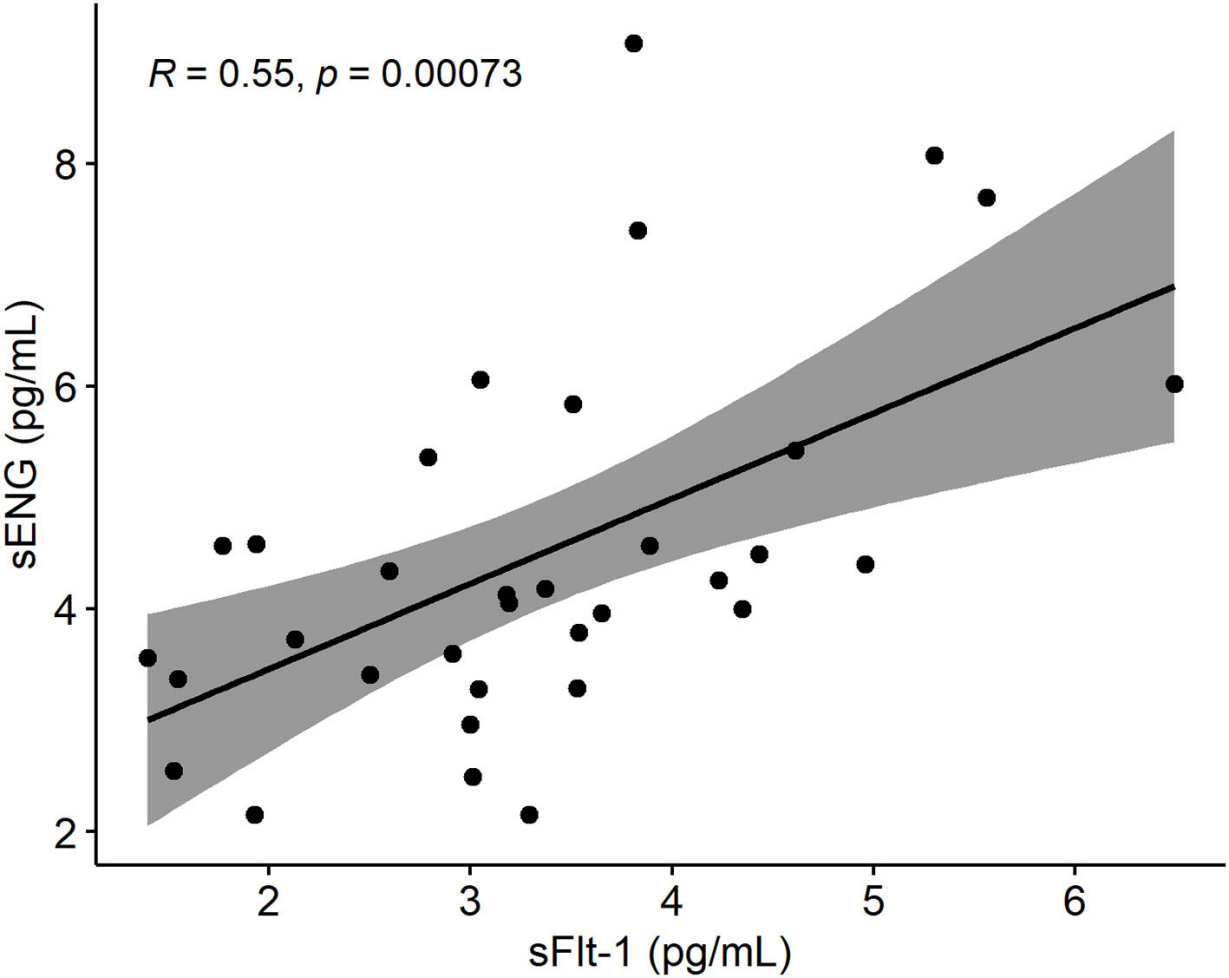
Spearman's rank correlation plot between sENG and sFlt-1 measurements of sera drawn from pregnant women within 70 and 98 gestational days. Gray around the linear regression line (y = 0.7651x + 1,931.4) indicates the 95% confidence region. sENG, soluble endoglin; sFlt-1, soluble fms-like tyrosine kinase-1.

Early pregnancy sFlt-1 and sENG serum levels were highly correlated (Figure 4). When sFlt-1 was replaced with sENG (model 1B) or both biomarkers were incorporated (model 1C), these alternative formulas showed equivalent properties in predicting PE as the model 1A (Tables 4, 5).

### Placental *FLT1* Rs4769613 T/C Genotype Data Improve PE Prediction Models

We explored the added value of placental *FLT1* rs4769613 T/C, confidently associated with late-onset PE (17, 18), in the PE prediction models excluding (2A–2C) or including (3A−3C) the parity data (Figures 3A,B; Tables 4, 5; Supplementary Table S3). The overall PE prediction accuracy in early pregnancy was the highest when combining gestational age-adjusted PTX3, sFlt-1, sENG, and ADAM12 measurements with the parity and placental *FLT1* rs4769613 additive genotype data [model 3C: AUC 0.969 (95%CI 0.895–1.000)]. This model yielded a correct “rule in” or “rule out” PE in 29 out of 31 cases (93.5%; 95%CI 79.3–98.2%). In the series of models 3A–3C and model 2A, none of the true PE cases were missed. Models 3A–3C yielded only two false-positive PE predictions for pregnancies that remained normotensive. Taken together, including placental *FLT1* rs4769613 T/C genotype data further improved the specificity of PE prediction from 80% to 94.7% (model 1A vs. 3A, model 1C vs. 3C, Table 5).

### 6PLEX Assay Data in Mid-pregnancy Are Moderately Informative for PE Prediction

The development of PE prediction models using 6PLEX assay measurement data in mid-pregnancy (100–182 g days) was less informative compared to early pregnancy (Figure 3C, Tables 4, 5, Supplementary Table S3). The best performing model selected by the stepwise logistic regression model combined gestational age-adjusted PlGF measurements with parity data [Model 4: AUC 0.784 (95%CI 0.634–0.912)]. In total, 29 of 39 pregnancies [74.4% (95%CI 58.9–85.4)] received correctly “ruled in” or “ruled out” PE development. Including genotype data of the placental variant *FLT1* rs4769613 T/C (model 5) did not improve the prediction [accuracy 71.9% (95%CI 54.6–84.4)].

## Discussion

We have recently developed a maternal serum-based 6PLEX assay implemented on the Luminex^®^ xMAP platform that measures six PE biomarkers in a single test tube and has shown its potential as an informative screening test (prediction accuracy of 96.5%) for PE prediction during the second half of pregnancy (15). This study demonstrated that 6PLEX assay measurements of serum samples collected in early pregnancy are also informative for developing sensitive and accurate PE prediction models applicable already during 70–98 g days (Figure 3, Table 5, Supplementary Table S3). Further innovative aspects in the study were the exploitation of an unbiased machine learning approach to select statistically most significant PE prediction models (22–24), adjusting biomarker measurements for the gestational day at sampling, and incorporation of the placental genotypes of the *FLT1* rs4769613 T/C variant, confidently associated with PE susceptibility (17, 18). The prediction model combining gestational age-adjusted 6PLEX measurements of PTX3, sFlt-1, sENG, and ADAM12 with parity and placental *FLT1* rs4769613 T/C genotype data yielded a correct prediction of PE in 93.5% of analyzed cases with no false-negative predictions.

This is the first time a PE prediction model incorporated a genetic risk factor to be combined with maternal serum biomarker measurements and clinical data into a disease risk prediction algorithm. The proposed placental *FLT1* rs4769613 T/C genotyping that increased the predictive accuracy from 88.2% to 93.5% (Figure 3, Table 5) is a novel entry into the currently protein-based biomarker-ruled PE prediction landscape. The utility of the non-invasive prenatal screening (NIPS) approach that is based on cell-free fetal DNA (cffDNA) is rapidly developing, currently focusing on detecting large chromosomal aberrations in the fetus (25, 26). However, there are already available technological solutions that allow single gene variant detection using the cffDNA (27). Thus, using cffDNA to screen the placental *FLT1* rs4769613 T/C genotypes to be incorporated into the PE prediction models may soon be a feasible approach. In this perspective, the most rational solution would be the inclusion of this variant in gene panels developed for NIPS targeting fetal single gene defects. Simultaneous blood sampling and screening of pregnant women for fetal chromosomal and monogenic conditions, as well as for their risk to develop PE, is also a cost-effective, time-saving, and patient-friendly approach. Although currently available NIPS-based tests for fetal monogenic disorders cost hundreds of euros and are expensive for screening purposes, the prices are expected to drop in long run with possible new competitive technological and cost-effective solutions arriving on the market.

The developed 6PLEX assay-based PE prediction models performed at least equally or even better than most PE prediction algorithms that are currently implemented in early pregnancy (12, 13). One of the most widely used and validated algorithms for detecting PE is based on combining maternal factors, uterine arteries pulsatility index (UtA Pl), mean arterial pressure (MAP), maternal serum PlGF, and/or PAPP-A (10). This algorithm can predict 90% of PE onset <32 g weeks, 75% of preterm PE (<37 g weeks), but only <50% for term PE cases. As three of four PE pregnancies develop ≥37th g week, this algorithm has its limitation. Further shortcomings of the currently used solution include a high false-positive rate (10, 28). In addition, the requirement of certified costly apparatus and trained personnel for the measurement of UtA PI is not a routine procedure in the management of pregnant women in many countries (11).

Combined data from this study and our previous report (15) provide strong evidence that placental biomarkers circulating in maternal serum have individual gestational dynamics that must be considered in PE prediction models. 6PLEX data are consistent with the published observations on the measurements of the same biomarkers using conventional single marker assays (29, 30). There is enough accumulated evidence that during each gestational period, the set of maternal serum metabolites reflecting placental and/or maternal pathology varies. For example, well-established PE biomarkers of late pregnancy sFlt-1 and PlGF (8, 11, 12, 16) show early pregnancy serum levels that are individually not equivocally discriminative for the PE onset during the 3rd trimester (Figures 1, 2). In contrast, circulating levels of PTX3 in early pregnancy were shown as an informative biomarker for later PE development in this study and by others (31, 32).

The development of a potentially applicable predictive model using mid-pregnancy serum biomarker data has appeared to be a challenging task. In the current study, 6PLEX measurements of samples collected at 100–182 g days were insufficiently informative to develop reliable PE prediction models for mid-pregnancy (accuracy <75%). Further studies are needed to discover maternal serum biomarkers that are specific to the 2nd trimester of pregnancy.

Possible limitations of our study have to be acknowledged, including the moderate sample size that does not cover the real-life variability of pregnancy scenarios (e.g., twin pregnancies) and the narrow demographic origin of patients. Large-scale follow-up studies in independent pregnancy cohorts, both retrospective and prospective, are needed to validate the developed PE prediction models and evaluate their performance in clinical practice.

## Conclusion

Despite all the research efforts and clinical advances, PE has remained a severe and rather common pregnancy complication with significant harm to maternal and perinatal morbidity. Timely surveillance and management of at-risk patients is the key approach to reducing its morbidities and most severe outcomes. Therefore, further early prediction tools, especially for late-onset PE, are needed to combine reliable screening performance with easily applicable protocols that do not need expensive infrastructure and special training and will be accessible to a large number of clinical centers. The developed model opens new horizons for first-trimester PE screening, combining the easily standardizable 6PLEX assay with routinely collected antenatal care data and resulting in high sensitivity and specificity of the test.

## Data Availability Statement

The original contributions presented in the study are included in the article/Supplementary Material, further inquiries can be directed to the corresponding author.

## Ethics Statement

The studies involving human participants were reviewed and approved by Ethics Committee of Human Research of the University Clinic of Tartu, Estonia (permissions no. 221/T-6, 17.12.2012 and 286/M-18, 15.10.2018). The patients/participants provided their written informed consent to participate in this study.

## Author Contributions

Conception: ML. Design: KRa, KRu, and ML. Provision of study materials: ML and KRu. Clinical data collection: KRu and EH. Experimental conduct: KRa and TK. Experimental guidance: KK and ML. Data analysis: KRa, OA, TK, and KF. Data interpretation: KRa, KRu, OA, TK, EH, KK, KF, and ML. Manuscript writing: KRa and ML. Critical reading and commenting on the article: KRu, EH, TK, KK, OA, and KF. All authors contributed to the article and approved the submitted version.

## Funding

ML and KRu, EU European Regional Development Fund (project no. 3.2.0701.12-0047); ML, Estonian Research Council (grant no. IUT34-12, PRG1021, EAG112); KF, Estonian Research Council (project no. PRG1197).

## Conflict of Interest

The authors declare that the research was conducted in the absence of any commercial or financial relationships that could be construed as a potential conflict of interest.

## Publisher's Note

All claims expressed in this article are solely those of the authors and do not necessarily represent those of their affiliated organizations, or those of the publisher, the editors and the reviewers. Any product that may be evaluated in this article, or claim that may be made by its manufacturer, is not guaranteed or endorsed by the publisher.
