# Supplementary material for: Novel Early Pregnancy Multimarker Screening Test for Preeclampsia Risk Prediction
Source: Front Cardiovasc Med. 2022 Jul 27;9:932480. doi: 10.3389/fcvm.2022.932480 (PMC9363612; doi:10.3389/fcvm.2022.932480)
Supplement: Supplementary file 1 [file Data_Sheet_1.pdf]

SUPPLEMENTARY MATERIALS

NOVEL EARLY PREGNANCY MULTIMARKER SCREENING TEST FOR PREECLAMPSIA RISK PREDICTION

Running title: Early multimarker test for preeclampsia

Kaspar Ratnik[1,2], Kristiina Rull[1,3,4], Oliver Aasmets[5], Triin Kikas[1], Ele Hanson[3,4], Kalle Kisand[6], Krista Fischer [5,7], Maris Laan[1]*

[1] Department of Biomedicine, Institute of Biomedicine and Translational Medicine, University of Tartu, Tartu, Estonia

[2] SYNLAB Eesti OÜ, Tallinn, Estonia

[3] Women's Clinic, Tartu University Hospital, Tartu, Estonia

[4] Department of Obstetrics and Gynaecology, Institute of Clinical Medicine, University of Tartu, Tartu, Estonia

[5] Estonian Genome Centre, Institute of Genomics, University of Tartu, Tartu, Estonia

[6] Department of Internal Medicine, Institute of Clinical Medicine, University of Tartu, Tartu, Estonia

[7] Institute of Institute of Mathematics and Statistics, University of Tartu, Tartu, Estonia

***Correspondence:** Maris Laan, PhD, Institute of Biomedicine and Translational Medicine, University of Tartu, Tartu 50411, Estonia. E-mail: maris.laan@ut.ee

SUPPLEMENTARY METHODS

Happy Pregnancy study cohort

Female participants were recruited at the Women's Clinic, Tartu University Hospital (TUH) March 2013 – August 2015 in the framework the of HAPPY PREGNANCY research project (full name: 'Development of novel non-invasive biomarkers for fertility and healthy pregnancy'). The study and the subsequent analysis of collected materials has been approved by the Research Ethics Committee of the University of Tartu, Estonia (permissions no 221/T-6, 17.12.2012; 286/M-18, 31.01.2018). The project aimed to test and evaluate the clinical relevance of serum markers and DNA polymorphisms in molecular diagnostics in reproductive medicine. All participants of this study were of white European ancestry and living in Estonia and they had signed written informed consent.

Informed consent was obtained from every subject at the first antenatal visit. For every participant, longitudinal anthropometric, epidemiological (three questionnaires across gestation), clinical data and biological material throughout the pregnancy and at the delivery were collected. At every clinical visit, all study participants had been monitored for their weight gain, arterial blood pressure dynamics, symptoms of proteinuria and signs of PE. The data about the further course and pregnancy outcome including fetal parameters were obtained from medical documentation.

All blood samples from the 'Happy Pregnancy' study participants were collected into Becton Dickinson Vacutainer® SST™ Serum Separation Tubes containing spray-coated silica and a polymer gel (Becton Dickinson Company, Franklin Lake, NJ, USA). Serum was separated in the service laboratory (United Laboratories, Tartu University Hospital) using routine procedures according to manufacturer's instructions (centrifugation at 1,800 g for 10 min at room temperature, RT). Serum samples were kept at -80°C before further aliquoting and subsequent analysis.

Clinical diagnosis of preeclampsia

Clinical signs at routine antenatal visits alerting to PE were considered single measurements of increased blood pressure (systolic blood pressure, SBP ≥140, diastolic blood pressure, DBP ≥90 mmHg), signs of proteinuria (amount of protein in urine ≥0.3 g/l) or development of gestational hypertension after 20th week of pregnancy. Hypertension was confirmed when the abnormal measurements were obtained in sitting position in two occasions at least 4 hours apart while the patient had been resting for at least 15 minutes. PE was diagnosed at the onset of both

hypertension (SBP ≥140 mmHg; DBP ≥90 mmHg) and proteinuria (2+ protein or greater on dipstick urinalysis, ≥300 mg of protein per 24-h urine collection). Alternative to increased urinary protein, other relevant clinical symptoms were considered, such as headache resistant to analgesics or visual disturbances, epigastric pain, severe edema, and oliguria after 20 g.week.

Luminex xMAP® technology

xMAP® suspension array technology is based on polystyrene beads with a diameter of 5.6 μm that are internally dyed with various ratios of two spectrally distinct fluorophores (Luminex® Corporation, Austin, TX, USA). As a result, an array of up to 500 different bead sets with specific absorption spectra is created. Various biological molecules, such as individual oligonucleotide probes, proteins or antibodies, can be coupled to alternative sets of beads. These sets are combined to a suspension array and due to their unique absorption spectra, it is possible to measure simultaneously up to 500 different probes in a single multiplex reaction. The technology is capable of performing both protein- and nucleic acid-based analyses, enabling both quantitative protein assays and qualitative DNA-based detection assays. Reference: https://www.luminexcorp.com/research/our-technology/xmap-technology/

Reagents for the Luminex® sandwich immunoassays

Luminex® xMAP based multiplexed assay was targeted to six previously established maternal serum predictive biomarkers for preeclampsia (**Table S2**) (15). Luminex® magnetic microspheres (#MC100) and antibody coupling kit for covalent linking of antibodies and microspheres (Antibody Coupling Kit, #40-50016) were purchased directly from Luminex® Corporation (Austin TX, USA). Capture and detection antibodies, and reference proteins were purchased from R&D Systems (Minneapolis, MN, USA) (**Table S2**). Antibodies were covalently linked to the surface of the fluorescently labeled Luminex® microspheres according to the manufacturer`s protocol and kept in dark at +4 °C until use. Stock solutions of each reference protein were prepared according to respective manufacturer`s instruction. Stock solutions were further diluted in 1% BSA (#810685; Merck KGaA, Darmstadt, Germany) to prepare the 10X Standard-8 solutions of either individual or multiplexed reference proteins, corresponding to 10-fold higher concentration of the maximum expected value of each analyte. Prior each immunoassay experiment, fresh working solutions the Standard-8 and its two-fold serial dilutions (Standard-7 and subsequent dilutions representing decreasing concentrations) were prepared in General Assay Diluent (GAD; #620; ImmunoChemistry Technologies LLC, Minnesota, USA). GAD represents a mammalian protein based immunoassay additive that

served as a blank measurement (Standard-1) for each analyzed biomarker. Antibodies, stocks and 10X Standard-8 solutions of reference proteins were kept at -80 °C.

Luminex® sandwich immunoassay protocol

All incubations during the immunoassay protocol were carried out at RT using a microplate shaker with rotational movements (speed set to 500 rpm; #4625, Barnstead Lab-Line, MA, USA). At the end of each incubation step, plates were placed on the Luminex® Magnetic Plate Separator (#CN-0269-01) for 1 min to bring all magnetic microspheres to the bottom of each well, followed by supernatant aspiration. All washing steps between incubations used 100 µL of WB per cell. Prior immunoassay, 96-well round bottom microplates (#734-1642 Corning, NY, USA) were treated for 10 min with Blocking Buffer (BB; 100 µL per well; 1% BSA, 0.02% Tween-20 in PBS at pH 7.4). After removal of BB, capture antibody coupled microbead solution prepared in WB (50 µL per well containing 2,500 beads of each analyte) and tested samples (`Standards8-1` or sera diluted in GAD; 50 µL/well) were pipetted to microplates. The mixture was incubated together for 2 h, followed by a washing step. Next, a biotinylated monoclonal or polyclonal detection antibody (or mixture of antibodies for multiplex assays; 100 µL/ well) diluted in WB was incubated for 1 h with a subsequent washing step. For the labeling of the immunoassay with a fluorescent reporter, incubation with streptavidin-phycoerythrin conjugate (100 µL of 1 µg/mL solution in WB; #PZPJ39S, Europa Bioproducts Ltd, Cambridge, UK) was applied for 30 min, followed by two rounds of washings. Finally, microbeads were resuspended in 75 µL of WB, a minimum of 50 biomarker-specific beads were collected from each well and analyzed on Luminex® MAGPIX analyzer (Luminex® Corporation, Austin TX, USA) using weighted 5-parameter logistic model implemented in Luminex® xPONENT 4.1 software (Luminex® Corporation, Austin TX, USA).

Placental sampling and preparations for *FLT1* rs4769613 genotyping

The sampling was conducted within 1 h after caesarean section or vaginal delivery, placentas kept at +4ºC meanwhile. A full-thickness blocks of 2 cm were taken from the middle region of each quadrant of the placenta. The excision site was chosen away from the umbilical cord insertion site, large vessels and any visible or palpable infarction, hematoma, or damage. Samples were washed with 1xPBS (phosphate-buffered saline, pH~7.4) to remove maternal blood. Sample was placed into a dry tube and stored at -80°C until DNA extraction. DNA from all of the placental samples was extracted using the NucleoSpin Tissue kit (MACHEREY-NAGEL GmbH & Co. KG, Düren, Germany) according to the manufacturer's protocol (18).

SUPPLEMENTARY TABLES

Supplementary Table S1. Clinical characteristics and preeclampsia risk factors of analyzed pregnancies.

Parameter	Analyzed index gestations	
	Controls with no PE	PE pregnancies
Number of subjects	31	22
Maternal age (years)	28.5 ± 5.1<br>28 (18 – 39)	28.0 ± 5.2<br>26.5 (20 – 39)
Maternal BMI at conception (kg/m2)	25.7 ± 4.8<br>25.5 (16.1 – 38.9)	26.8 ± 6.2<br>26.3 (18.0 – 42.9)
Mean arterial blood pressure at first visit (mm Hg)	84.3 ± 7.7<br>85.0 (66.0 – 96.6)	86.9 ± 9.1<br>86.6 (73.3 – 103.3)
Gravidity (n)	1.9 ± 1.3<br>2 (1 – 6)	1.7 ± 1.2<br>1 (1 – 4)
Smoking during pregnancy	1	1
Maximum SBP (mm Hg)	128.9 ± 12.1<br>125 (100 – 50)	159.1 ± 11.6*<br>160 (135 – 177)
Maximum DBP (mm Hg)	80.2 ± 12.5<br>80 (60 – 100)	101.6 ± 8.9*<br>100 (80 – 122)
Nulliparity (n), %	16 (52%)	17(77%)
Obesity, BMI >30 kg/m2 (n)	5 (16%)	6 (27%)
Previous PE or hypertensive disorder/multiparous women (n)	4/12	3 /5
Pre-existing hypertension	2	0
Family history of gestational hypertension and PE	0	2
Pre-existing diabetes / gestational diabetes	0/3	1/0
Pregnancy after IVF	0	1
Hypertension or single hypertensive measurement during the pregnancy (n), %	15 (48%)	22 (100%)*
Proteinuria during the pregnancy (n), %	1 (3%)	22 (100%)*
Gestational age at birth (days)	279.6 ± 11.3<br>283 (236 – 292)	257.0 ± 20.8*<br>261.5 (199 – 284)
Preterm delivery <37 gest. weeks (n)	1 (3%)	7 (32%)*
Newborn weight (g)	3579 ± 581<br>3558 (1510 – 4442)	2643 ± 728*<br>2642 (814 – 4274)
SGA newborn (n)	3 (10%)	9 (41%)*
Newborn's sex (F/M)	16/15	10/12
Labor induction (n)	3 (10%)	11 (50%)*

Delivery mode (vaginal/C-section)	27/4	15/7*

Data are presented as mean ± standard deviation and median (min-max values), except where indicated differently.
*Statistically significant difference ($p<0.05$). Chi-squared test for categorical and Wilcoxon rank-sum test for non-categorical variables was applied.
Diagnosis of SGA was assigned at the delivery based on national guidelines (20).

BMI, body mass index; C-section, Caesarean section; F/M, female/male; Gravidity, total number of pregnancies including index pregnancy; IVF, *in vitro* fertilization; n, number of subjects; Nulliparity, no previous births; PE, preeclampsia; SBP/DBP, systolic/diastolic blood pressure; SGA, small for gestational age

Supplementary Table S2. Antibodies and reference proteins utilized in the development of Luminex® multiplex assay.

Biomarker	Uniprot #	Standard protein	Capture antibody	Detection antibody
ADAM12	O43184	Recombinant Human ADAM12, CF, #4416-AD-020 (R&D Systems)	Anti-ADAM Monoclonal Mouse IgG Clone #632525 (R&D Systems)	Biotinylated Anti-human ADAM12 Antibody Sheep IgG BAF4416 (R&D Systems)
Leptin	P41159	Recombinant Human Leptin, CF, #398-LP-01M (R&D Systems)	Anti-Leptin Monoclonal Mouse IgG Clone #44802 (R&D Systems)	Human Leptin Biotinylated Antibody Monoclonal Mouse IgG BAM398 (R&D Systems)
PTX3	P26022	Recombinant Human Pentraxin 3/TSG-14, CF, #1826-TS-025/CF (R&D Systems)	Anti-Pentraxin 3 Monoclonal Mouse IgG Clone #247911 (R&D Systems)	Human Pentraxin 3 Biotinylated Antibody Polyclonal Goat IgG BAF1826 (R&D Systems)
PlGF	P49763	Recombinant Human PlGF, CF, #264-PG-010/CF (R&D Systems)	Anti-PlGF Monoclonal Mouse IgG Clone #37203 (R&D Systems)	Human PlGF Biotinylated Antibody Polyclonal Goat IgG BAF264 (R&D Systems)
sENG	P17813	Recombinant Human Endoglin/CD105, CF, #1097-EN-025/CF (R&D Systems)	Anti-ENG Monoclonal Mouse IgG Clone #166713 (R&D Systems)	Human Endoglin Biotinylated Antibody Polyclonal Goat IgG BAF1097 (R&D Systems)
sFlt-1	P17948	Recombinant Human VEGF R1/Flt-1 Fc Chimera, CF, #321-FL-050/CF (R&D Systems)	Anti-Flt1 Monoclonal Mouse IgG Clone #49560 (R&D Systems)	Antigen Affinity-purified Human Flt-1 Polyclonal Goat IgG Biotinylated BAF321 (R&D Systems)

ADAM12, disintegrin and metalloproteinase domain-containing protein 12; PlGF, placental growth factor; sFlt-1, soluble fms-like tyrosine kinase-1; PTX3, pentraxin-related protein 3 *alias* TNF-inducible gene 14 protein; sENG, soluble endoglin; sFlt-1, soluble fms-like tyrosine kinase-1

Supplementary Table S3. Formulas, coefficients and thresholds for preeclampsia prediction models.

Model acronym	Formula	Threshold value	Coefficients								
			a-interc	b-sFlt-1	c-ADAM12	d-PTX3	e-sENG	g- PlGF	h-parity	i-rs4769613 T/C	j-g.days
1A	p(i) = 1/(1 + *e*^(-a -b x log2([sFlt-1]) -c x log2([ADAM12]) -d x log2([PTX3]) – h x parity -j x gest.age)	0.243	-15.859	1.778	-1.703	-1.937			-4.103		0.145
1B	p(i) = 1/(1 + *e*^(-a -c x log2([ADAM12]) -d x log2([PTX3]) - e x log2([sENG]) – h x parity -j x gest.age)	0.243	2.608		-1.535	-1.642	0.459		-3.158		0.134
1C	p(i) = 1/(1 + *e*^(-a - b x log2([sFlt-1]) -c x log2([ADAM12]) -d x log2([PTX3]) - e x log2([sENG]) – h x parity -j x gest.age)	0.244	-16.454	1.906	-1.561	-1.911	-0.363		-4.123		0.132
2A	p(i) = 1/(1 + *e*^(-a -b x log2([sFlt-1]) -c x log2([ADAM12]) -d x log2([PTX3]) – i x rs4769613 T/C -j x gest.age)	0.182	-5.524	1.969	-3.599	-1.710				-2.227	0.144
2B	p(i) = 1/(1 + *e*^(-a -c x log2([ADAM12]) -d x log2([PTX3]) - e x log2([sENG]) – i x rs4769613 T/C -j x gest.age)	0.208	14.631		-2.647	-1.146	-0.237			-1.416	0.087
2C	p(i) = 1/(1 + *e*^(-a - b x log2([sFlt-1]) -c x log2([ADAM12]) -d x log2([PTX3]) - e x log2([sENG]) – i x rs4769613 T/C -j x gest.age)	0.356	-11.230	3.519	-3.808	-2.072	-2.799			-3.333	0.114
3A	p(i) = 1/(1 + *e*^(-a -b x log2([sFlt-1]) -c x log2([ADAM12]) -d x log2([PTX3]) – h x parity - i x rs4769613 T/C -j x gest.age)	0.308	-2.776	2.865	-3.056	-1.871			-20.721	-1.911	0.174
3B	p(i) = 1/(1 + *e*^(-a -c x log2([ADAM12]) -d x log2([PTX3]) - e x log2([sENG]) – h x parity - i x rs4769613 T/C -j x gest.age)	0.261	28.304		-1.549	-1.186	-0.196		-19.146	-1.266	0.054
3C	p(i) = 1/(1 + *e*^(-a - b x log2([sFlt-1]) -c x log2([ADAM12]) -d x log2([PTX3]) - e x log2([sENG]) – h x parity - i x rs4769613 T/C - j x gest.age)	0.412	-8.665	3.907	-2.718	-2.075	-1.850		-20.777	-2.464	0.136
4	p(i) = 1/(1 + *e*^(-a - g x log2([PlGF]) – h x parity - j x gest.age)	0.463	-0.744					0.496	0.622		-0.035
5	p(i) = 1/(1 + *e*^(-a - g x log2([PlGF]) – h x parity- i x rs4769613 T/C - j x gest.age)	0.415	-2.162					0.456	1.018	-0.089	-0.024

For every patient, application of the formula generates the prediction value (p(i)). The estimated (p(i)) equal or superior to a threshold value indicates that the subject will develop PE during III trimester. The estimated (p(i)) inferior to a threshold value indicates that the subject will not develop preeclampsia during III trimester. The caret (^) refers to the exponentiation operator.

ADAM12, disintegrin and metalloproteinase domain-containing protein 12; PlGF, placental growth factor; PTX3, pentraxin-related protein 3; sENG, soluble endoglin; sFlt-1, soluble fms-like tyrosine kinase-1; g.days, gestational age in days
